# The new preservative-free ophthalmic formulation of bilastine 0.6% preserves the ocular surface epithelial integrity in a comparative in vitro study

**DOI:** 10.1038/s41598-024-59190-5

**Published:** 2024-04-26

**Authors:** Eider Arana, Ana Gonzalo, Noelia Andollo, Felipe Goñi-de-Cerio, Paloma Gómez-Fernández, Clarisa Salado, Gonzalo Hernández, Tatiana Suárez-Cortés

**Affiliations:** 1 Research, Development and Innovation Department (R&D+I Department), Faes Farma, Av. Autonomía 10, 48940 Leioa, Bizkaia Spain; 2 Department of Cell Biology and Histology, School of Medicine and Nursing, University of the Basque Country, Leioa, Spain; 3 Biobizkaia Health Research Institute, Barakaldo, Spain; 4 Biotechnology Area, GAIKER Technology Centre, Basque Research and Technology Alliance, Zamudio, Spain; 5 Innoprot SL, Bizkaia Technology Park, Derio, Bizkaia Spain

**Keywords:** Medical research, Preclinical research, Conjunctival diseases, Corneal diseases

## Abstract

Allergic conjunctivitis (AC) is the most common form of allergic eye disease and an increasingly prevalent condition. Topical eye drop treatments are the usual approach for managing AC, although their impact on the ocular surface is not frequently investigated. The aim of this study was to perform a comparative physicochemical characterization, and in vitro biological evaluations in primary conjunctival and corneal epithelial cells of the new multidose preservative-free bilastine 0.6% and main commercially available eye drops. MTT assay was used to measure cell viability; oxidative stress was analyzed with a ROS-sensitive probe; and apoptosis was evaluated monitoring caspase 3/7 activation. Differences in pH value, osmolarity, viscosity and phosphate levels were identified. Among all formulations, bilastine exhibited pH, osmolarity and viscosity values closer to tear film (7.4, 300 mOsm/l and ~ 1.5–10 mPa·s, respectively), and was the only phosphates-free solution. Single-dose ketotifen did not induce ROS production, and single-dose azelastine and bilastine only induced a mild increase. Bilastine and single-dose ketotifen and azelastine showed high survival rates attributable to the absence of preservative in its formulation, not inducing caspase-3/7-mediated apoptosis after 24 h. Our findings support the use of the new bilastine 0.6% for treating patients with AC to preserve and maintain the integrity of the ocular surface.

## Introduction

Ocular allergy is a pathology that includes several clinical conditions that can be considered as hypersensitivity disorders of the ocular surface^1,2^. Allergic conjunctivitis (AC), the most common form of allergic eye disease, is mainly caused by an allergen-induced inflammatory response that is characterized by itching, conjunctival hyperemia, excessive tearing, and conjunctival and eyelid swelling^3–5^. The most frequent clinical forms of ocular allergy are seasonal and perennial AC, which are predominantly caused by an IgE-mediated hypersensitivity reaction^6,7^. The distressing signs and symptoms of AC may cause extreme discomfort and can potentially be vision threatening, negatively impacting the quality of life of affected patients^6,8^.

The prevalence of ocular allergy has been increasing worldwide for the past several decades and, at present, approximately 20% of the world population is affected by some form of allergy^6,9^. It is reported that up to 40–60% of allergic patients have ocular symptomatology^10^. As the prevalence of AC rises, the development of novel drugs for the prevention and treatment of this condition also increases.

Optimal management of AC is aimed at providing relief of symptoms and pharmacologic suppression of inflammatory responses. Systemic or topical antihistamines, mast cell stabilizers, and dual activity agents are considered first-line management strategies for AC^11^. Other treatment options include nonsteroidal anti-inflammatory drugs, steroids, allergen-specific immunotherapy, and some off-label treatments^11–13^.

Topical pharmacologic treatments, such eye drop instillation, are considered the most widely preferred and safe route of ocular drug administration in the treatment of AC^13–15^. In cases of isolated ocular symptoms, the use of topical ocular antihistamines is the treatment of choice. Ophthalmic solutions are available for multidose or single-dose administration, although most commercially available medications are multidose eye drop formulations^16^. In order to maintain sterility throughout its intended length of use and prevent ocular infections from contaminated eye drops, the addition of preservatives in multidose formulations is crucial. However, preservatives can induce ocular surface damage. The most commonly used preservative is benzalkonium chloride (BAC), present in approximately 70% of ophthalmic formulations, which is well known to cause tear film instability, toxicity to conjunctival and corneal epithelial cells, and to induce ocular surface inflammation^17,18^. Consequently, to avoid the adverse effects caused by the inclusion of preservatives, most of the topical ophthalmic medications have become progressively available in preservative-free formulations. Preservative-free multidose eye drops rely on a filtering system to stop the entry of microorganisms^19^. This technology offers the advantages of a preservative-free eye drops, as well as the benefits of a multidose format, including an improved use and an enhanced compliance.

Recently, a new once-daily multidose preservative-free ophthalmic formulation of bilastine 0.6% containing sodium hyaluronate has been developed for the topical treatment of AC^20^. Bilastine is a second-generation H_1_-antihistamine recommended for the symptomatic treatment of seasonal or perennial allergic rhinoconjunctivitis and urticaria in adults and children^21,22^. The efficacy, safety and tolerability of the new ophthalmic formulation has been demonstrated in Phase II and Phase III clinical trials^20,23,24^. Bilastine displays good efficacy with a rapid onset of action, a prolonged effect, and good tolerability with minimal sedative properties^21–23,25,26^.

Although there are a large number of efficacy studies of topical ophthalmic formulations for the treatment of AC, research of their impact on the ocular surface is still lacking. In fact, long-term use of antiallergic eye drops may cause serious complications and side effects affecting the ocular surface that may be caused not only by preservatives but also by the active pharmaceutical ingredient (API) and/or vehicle in the topical formulation^27^. Hence, the aim of this study was to perform a comparative physicochemical characterization and in vitro biological evaluations in conjunctival and corneal epithelial cells of the new once-daily multidose preservative-free bilastine 0.6% formulation and the main commercially available eye drops (BAC-preserved and preservative-free formulations) commonly used for the treatment of AC.

## Materials and methods

### Eye drop formulations

The once-daily multidose preservative-free bilastine 0.6% formulation and eight preserved and preservative-free commercially available antiallergic eye drops evaluated in the present study are listed in Table 1. In order to avoid biased conclusions, a code number was assigned to all eye drops to keep the investigator unaware of the formulation tested. The code was composed of three letters (EDF, which stands for “Eye Drop Formulation”) followed by consecutive numbers, from one to nine.

**Table 1:** Antiallergic eye drops evaluated in the present study.

Code	Active component (% w/v)	Trade name	Delivery system	Mechanism of action	Preservative (% w/v)
EDF 1	Ketotifen fumarate (0.025%)	Zaditen® (Laboratorios Théa, Barcelona, Spain)	Single-dose	DUAL	PF
EDF 2	Azelastine hydrochloride (0.05%)	Tebarat® (Laboratorios Salvat, Barcelona, Spain)	Single-dose	DUAL	PF
EDF 3	Bilastine (0.6%)	Bilaxten® (Faes Farma, Bizkaia, Spain)	Multidose	AH	PF
EDF 4	Olopatadine hydrochloride (0.222%)	Pataday® (Alcon Laboratories, Texas USA)	Multidose	DUAL	BAC (0.01%)
EDF 5	Azelastine hydrochloride (0.05%)	Afluon® (Mylan Pharmaceuticals, Barcelona, Spain)	Multidose	DUAL	BAC (0.0125%)
EDF 6	Olopatadine hydrochloride (0.776%)	Pazeo® (Alcon Research, Texas, USA)	Multidose	DUAL	BAC (0.015%)
EDF 7	Levocabastine hydrochloride (0.05%)	Bilina® (Esteve Pharmaceuticals, Barcelona, Spain)	Multidose	AH	BAC (0.015%)
EDF 8	Olopatadine hydrochloride (0.1%)	Opatanol® (Novartis Farmacéutica, Barcelona, Spain)	Multidose	DUAL	BAC (0.01%)
EDF 9	Ketotifen fumarate (0.025%)	Zaditen® (Laboratorios Théa, Barcelona, Spain)	Multidose	DUAL	BAC (0.01%)

AH, H1-receptor antagonistic action; BAC, Benzalkonium chloride; DUAL, histamine H1 receptor antagonistic action and mast cell stabilization action; EDF, eye drop formulation; PF, preservative-free.

### Cell lines and culture conditions

Human Primary Conjunctival Epithelial Cells (HConEpiC, P10870, Innoprot, Bizkaia, Spain), Human Primary Corneal Epithelial Cells (HCEpiC, P10871, Innoprot, Bizkaia, Spain) and Human Primary Corneal Epithelial Cells (HCEpC) (H-6048, Cell Biologics, Campbell Park Drive, Chicago, Illinois, USA) were used. The first two cell lines were cultured on collagen coated flasks in Corneal Epithelial Cell Medium (CEpiCM, P60131, Innoprot, Bizkaia, Spain), containing 500 ml of Corneal Epithelial Cell basal medium, 25 ml of Fetal Bovine Serum, 5 ml of Epithelial Cell Growth Supplement and 5 ml of Penicillin/Streptomycin solution (Corning, 25-051-CI). The third cell line was cultured on collagen coated flasks in Epithelial Cell Medium (EpiCM, P60106, Innoprot, Bizkaia, Spain), containing 500 ml of Epithelial basal medium, 10 ml of Fetal Bovine Serum, 5 ml of Epithelial Cell Growth Supplement and 5 ml of Penicillin/Streptomycin solution; all of these components were included in kit P60106 of the epithelial cell medium EpiCM. All cell cultures were maintained at 37 °C in a humidified atmosphere with 5% CO_2_. The medium was changed every 2–3 days, and cells were subcultured when they reached 80–90% confluence. The cells were detached with trypsin/EDTA (T4174, Sigma-Aldrich, St. Louis, Missouri, USA), and the enzyme mixture inactivated with 10% fetal bovine serum (A5256701 Gibco/Thermo Fisher Scientific, Waltham, Massachusetts USA).

### Physicochemical characterization

All eye drop formulations were characterized according to various physicochemical properties, including pH, osmolarity, viscosity and phosphate levels, and were compared to human tear film characteristics.

The pH value was measured at room temperature on a calibrated S8 Seven2Go™ pro pH/Ion Meter (Mettler-Toledo, Barcelona, Spain) using a pH electrode InLab Micro Pro-ISM (30014096, Mettler-Toledo, Barcelona, Spain).

Osmolarity of the topical eye drops was measured using the Fiske 210 microsample osmometer (Advanced Instruments Inc., Norwood, Massachusetts, USA).

The viscosity was determined using Fungilab™ Smart Series L Model Rotational Viscometer (Fungilab S.A., Sant Feliu de Llobregat, Spain), equipped with a temperature probe and a small sample adapter.

Quantification of phosphate content was carried out by inductively coupled plasma atomic emission spectrometry (ICP-OES). Analyses were performed using a 5100 ICP-OES from Agilent Technologies (Santa Clara, California, USA). Since no specific guides are available to classify products according to their phosphate content, we established a classification based on the quantification of phosphates of three artificial tear preparations commercially available in Spain, known to contain different phosphate levels: Aquoral® (0.4% sodium hyaluronate, solution containing phosphates; 315 ppm PO_4_^3^), Lubristil® (0.15% sodium hyaluronate, solution not containing phosphates; 0.9 ppm PO_4_^3-^), and HYLO-Dual® (0.05% sodium hyaluronate and 2% ectoin; phosphate-free solution, < 0.3 ppm PO_4_^3−^). Based on their phosphate concentrations, the eye drops were classified into one of these categories: “with phosphates” (> 1.0 ppm PO_4_^3−^), “without phosphates” (0.3–0.9 ppm PO_4_^3−^), and “phosphates-free” (< 0.3 ppm PO_4_^3−^).

### Cytotoxicity assays

Cell viability was quantitatively evaluated in vitro in HConEpiC and HCEpiC after 24- and 72-h exposure at different serial dilutions of the eye drop formulations (% v/v), using the MTT reduction assay. This assay involves the conversion of the water-soluble yellow dye MTT [3-(4,5-dimethylthiazol-2-yl)-2,5-diphenyltetrazolium bromide] (M2128, Sigma-Aldrich, St. Louis, Missouri, USA) into water-insoluble purple formazan crystals by living cells, which determines mitochondrial activity related to the number of viable cells^28,29^.

The MTT viability assay was performed as previously described^29^, with some modifications. Briefly, cells (100 µL, 1 × 10^4^ cells/ml) were plated into 96-well tissue-culture flat bottom plates, treated with two-fold serial dilutions of the ophthalmic formulations (100 µl, 50% to 0.39% v/v concentrations), and incubated for 24 h and 72 h at 37 °C with 5% CO_2_. Sodium dodecyl sulfate (SDS, CAS No 151-21-3, L4509, Sigma-Aldrich, St. Louis, Missouri, USA) was used as positive control for cytotoxicity at different concentrations (400 μg/ml to 3.13 μg/ml; two-fold serial dilution), and cells maintained in fresh basal medium were used as negative control. Following 24-h incubation, the formulations were removed, and cells were washed with phosphate-buffered saline (PBS, 11666789001, Roche, Basel, Switzerland) and stained with MTT reagent. After 2-h incubation at 37 °C with 5% CO_2_, the MTT solution was removed, and the formazan precipitates were solubilized with 100 µl of dimethyl sulfoxide (DMSO, CAS No 67-68-5, D2650, Sigma-Aldrich, St. Louis, Missouri, USA) for 15 min at room temperature. The absorbance was measured using a spectrophotometer (Multiskan spectrum 1500, Thermo Fisher Scientific, Vantaa, Finland) at a wavelength of 540 nm. The results were expressed as a percentage of cell viability (%) relative to the untreated control cells.

### Oxidative stress assessment/ reactive oxygen species (ROS) production

The intracellular oxidative stress was analyzed in HCEpC cells using a ROS-sensitive probe: 5-(and-6-)-chloromethyl-2′,7′-dichlorodihydrofluorescein diacetate, acetyl ester (CM-H_2_DCFDA, CAS No 1219794-09-8, C6827, Life Technologies, Gaithersburg, Maryland, USA), which is a cell-permeable, nonfluorescent compound that is oxidized by hydroxyl radical, peroxynitrite, and other reactive oxygen species to a fluorescein derivative^30^.

**Figure 1:**
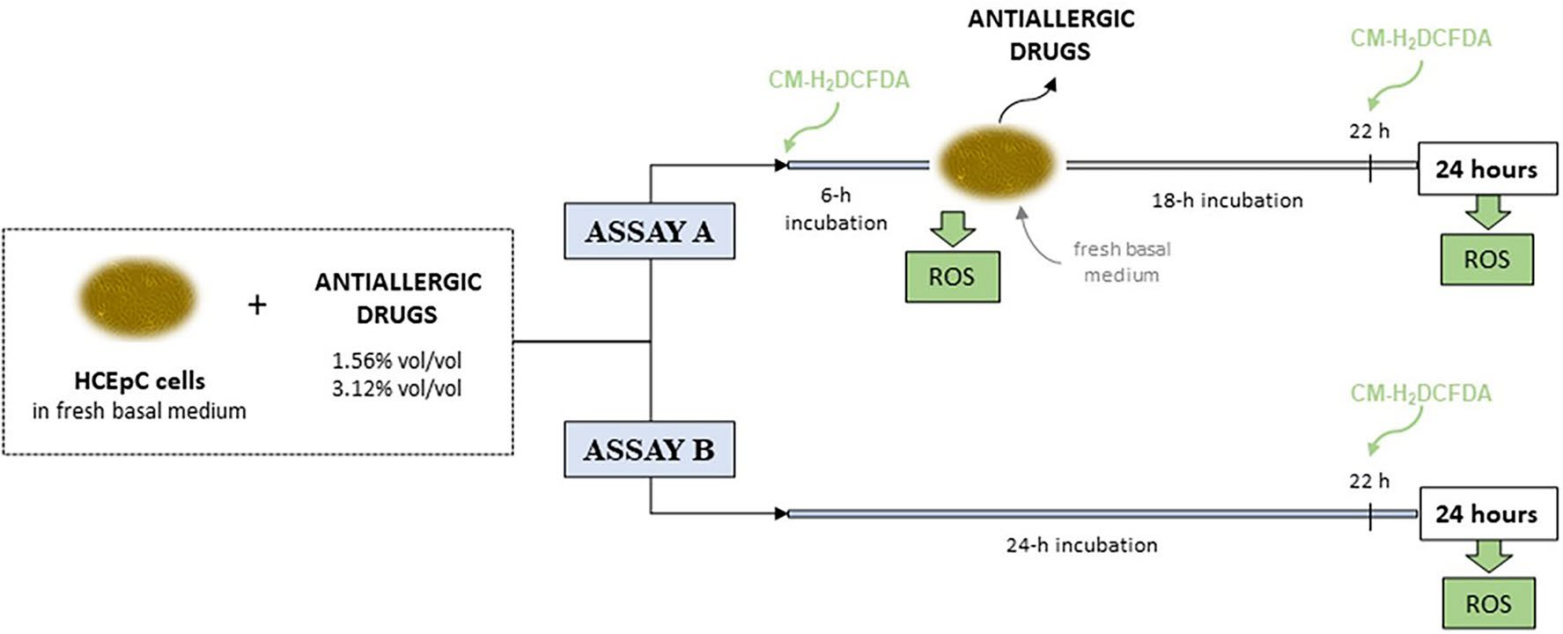
Oxidative stress methodology in Human Primary Corneal Epithelial Cells (HCEpC) using the Reactive Oxygen Species (ROS)-sensitive probe: 5-(and-6-)-chloromethyl-2′,7′-dichlorodihydrofluorescein diacetate, acetyl ester (CM-H_2_DCFDA). ASSAY A: Exposure of cells to ophthalmic formulations for 6 h, followed by an 18-h recuperation period. Cells were incubated with the eye drops for 6 h and washed to remove treatment; then, fresh basal medium was added for an additional 18-h incubation period. The probe was added both initially and after 22 h of incubation. ROS was measured at 6 h and at 24 h. ASSAY B: Overnight exposure of cells to ophthalmic formulations. The probe was added after 22 h of incubation. ROS was measured at 24 h.

The determination of ROS production was performed as previously described^30^, with some modifications. Briefly, HCEpC cells were seeded into 96-well flat bottom culture plates at a density of 3 × 10^4^ cells/well (100 µl/well) and incubated for 24 h at 37 °C with 5% CO_2_ in a humidified environment. Subsequently, two treatment assays were performed (Fig. 1). In Assay A, the ophthalmic formulations were removed after 6 h of treatment of cells, and fresh basal medium was added for an additional 18-h incubation period. In Assay B, incubation of cells with study formulations were maintained for 24 h. In both cases, cells were treated with two dilutions of the tested formulations (3.12% and 1.56%, v/v). For comparative purposes, a BAC stock solution (CAS No: 8001-54-5, B6295, Sigma-Aldrich, St. Louis, Missouri, USA) was prepared at 0.15 mg/ml, which corresponds to the highest concentration presented in the preserved tested formulations (see Table 1). From this stock solution, equivalent dilutions to those evaluated for the antiallergic formulations (3.12% and 1.56% v/v) were prepared in serum-free culture medium. These dilutions were used to investigate the effect of BAC on the same parameters previously assessed. Cells maintained in fresh basal medium were used as negative control, and cells treated with 250 µM H_2_O_2_ were used as positive control to measure ROS production.

In order to determine ROS production, 1 µM of CM-H_2_DCFDA was added to the cells at different time points, depending on the treatment assay. In assay A, the probe was added initially along with the formulations, and after 22 h; ROS was determined at 6 h, and after the recovery period, at 24 h. In Assay B, the probe was added after 22 h of incubation, and ROS was determined at 24 h. Cell fluorescence intensity was measured at 488 nm excitation and 530 nm emission using the CellInsight CX7 HCS platform (Thermo Fisher Scientific, Waltham, MA, USA).

### Apoptosis evaluation

The effect of formulations on cellular apoptosis was evaluated monitoring caspase 3/7 activation in HCEpC cells using the CellEvent™ Caspase 3/7 Green Detection Reagent (C10423, Invitrogen/ThermoFisher Scientific, Waltham, Massachusetts, USA). This reagent comprises the DEVD peptide conjugated to a nucleic acid–binding dye, which is fluorescent only when bound to nucleic acids. Because the DEVD peptide inhibits the ability of the dye to bind DNA, the reagent is intrinsically non-fluorescent. However, after activation of caspase 3/7, the DEVD peptide is cleaved enabling the dye binding to DNA and producing a bright green-fluorescent signal indicative of apoptosis.

Apoptosis evaluation was performed at two different time points, after 6 h of treatment of cells plus an additional 18-h incubation period, and after 24-h treatment. In both cases, cells were treated with two dilutions of the eye drop formulations (3.12% and 1.56% v/v). BAC dilutions (3.12% and 1.56% v/v) were also assayed in serum-free culture medium for comparison. At both time points, cells were stained with 5 μM of the CellEvent™ Caspase 3/7 Green Detection Reagent for 60 min at 37 °C in the dark. Then, cells were washed two times with PBS. The fluorescence was measured at 488 nm excitation and 530 nm emission using the CellInsight CX7 HCS platform. Positive and negative controls were included in every experiment. Specifically, treatment with 0.5 µM staurosporine was used as internal positive control of caspase activation, and cells maintained in fresh basal medium were used as negative control.

In addition, the fluorescent dye Hoechst 33342 (H1399, Life Technologies, Gaithersburg, Maryland, USA), which binds specifically to adenine–thymine-rich regions of DNA, was used to count HCEpC cells at the same time points. After treatment with two dilutions of the eye drop formulations (3.12% and 1.56% v/v) and the highest dose of BAC (3.12% v/v), HCEpC cells were stained with 5 µM of the fluorescent dye for 60 min at 37 °C, and were washed two times with PBS. The fluorescence intensity was measured at 460 nm excitation and 490 nm emission using the CellInsight CX7 HCS platform, and nine images per well were acquired using CellInsight CX7 High-Content Screening (HCS) Platform (20X objective). Apoptotic cells with activated caspase showed bright green spots and nuclei were stained in blue. Again, treatment with 0.5 µM staurosporine was used as internal positive control of caspase activation, and cells maintained in fresh basal medium were used as negative control.

Both procedures (apoptosis evaluation by fluorescent detection of caspase activation and quantification of cell density by Hoechst 33342 nuclear staining) were performed in parallel, under the same experimental conditions and in the same culture plates.

### Statistical analysis

For cytotoxicity data, the Kolmogorov–Smirnov test was used to confirm the normality of the distribution. Subsequently, data were analyzed by multivariate logistic regression models for HConEpiC and HCEpiC cells. Differences between treatments were determined using Student’s t-test and considered statistically significant at *p* < 0.05. The results in the cell viability studies were normalized respect to control cells. All statistical analyses were performed using SAS software, version 9.4 (SAS Institute Inc, Cary, NC, USA).

For oxidative stress data, statistical analyses were performed using the Prism 8.0 software (GraphPad, San Diego, CA). The Shapiro–Wilk test was used to assess normality in the distribution of data. Once confirmed that all data were normally distributed, statistical comparisons in ROS production, caspase 3/7 activation and Hoechst staining experiments were conducted using variance analysis (ANOVA) models and Dunnett´s multiple comparison test. ROS, caspase 3/7 and cell number results were normalized according to negative control (basal medium). All values were expressed as mean (± SD) of six separate wells. Statistical differences with respect to the negative control (basal medium treated cells) were indicated by *p < 0.1, **p < 0.01, ***p < 0.001 and ****p < 0.0001. Tukey’s multiple comparison test was used to compare the ophthalmic topical formulations with respect to EDF 3 formulation (bilastine 0.6%). Differences were considered statistically significant when #*p* < 0.05. Statistical analyses were performed with GraphPad Prism 9.0 (GraphPad Software, Inc., San Diego, CA).

## Results

### Physicochemical characterization

The results of pH, osmolarity, viscosity and phosphate levels of the eye drops included in the study are summarized in Table 2. The data obtained were compared to the physicochemical properties of the tear fluid. The average physiological pH of lacrimal fluid is 7.4^31,32^, its osmolarity is around 300 mOsm/l^33–36^, and its viscosity is in the range of 1.5–10 mPa·s^37,38^.

**Table 2:** Characterization of antiallergic eye drops evaluated in the study.

Code	Active component (% w/v)	pH [mean (SD)]	Viscosity (mPa·s) [mean (SD)]	Osmolarity (mOsm/L) [mean (SD)]	Phosphate concentration (PO_4_^3-^, ppm)	Classification according to phosphate concentration*
EDF 1	Ketotifen fumarate (0.025%)	5.31 (0.03)	1.2 (0.00)	245 (1.15)	< 0.6	Without phosphates
EDF 2	Azelastine hydrochloride (0.05%)	5.69 (0.01)	3.0 (0.00)	278 (1.00)	3.66	With phosphates
EDF 3	Bilastine (0.6%)	7.32 (0.02)	6.8 (0.12)	294 (1.15)	< 0.3	Phosphates-free
EDF 4	Olopatadine hydrochloride (0.222%)	7.11 (0.00)	1.5 (0.00)	308 (1.53)	3,213	With phosphates
EDF 5	Azelastine hydrochloride (0.05%)	5.90 (0.03)	2.5 (0.00)	282 (2.31)	< 0.6	Without phosphates
EDF 6	Olopatadine hydrochloride (0.776%)	7.10 (0.01)	27.7 (0.06)	331 (4.36)	1.8	With phosphates
EDF 7	Levocabastine hydrochloride (0.05%)	6.94 (0.00)	6.4 (0.06)	1038 (3.06)	9207	With phosphates
EDF 8	Olopatadine hydrochloride (0.1%)	7.11 (0.00)	1.2 (0.00)	295 (1.53)	3324	With phosphates
EDF 9	Ketotifen fumarate (0.025%)	5.39 (0.01)	1.4 (0.00)	245 (1.15)	< 0.6	Without phosphates

Data are presented as the mean ± SD (standard deviation).

EDF, eye drop formulation.

*Category according to the classification described in the Material and Methods section.

Regarding the pH parameter, the tested products EDF 1, EDF 2, EDF 5, and EDF 9 had acidic pH values in the range of 5.31–5.90; whereas EDF 3, EDF 4, EDF 6, EDF 7 and EDF 8 showed pH values around 7.0. Among these agents, EDF 3 showed the pH value closer to the physiological pH of tears (pH = 7.32).

Osmolarity values were close to tear osmolarity and ranged from 245 to 331 mOsm/l, except for EDF 7, an ophthalmic suspension showing a hyperosmolar value of 1038 mOsm/l. With regard to viscosity, all formulations, except for EDF 6 (27.7 mPa·s), had values between 1.2–6.8 mPa·s (< 10 mPa·s). Among them, EDF 3 and EDF 7 showed the highest viscosity values (6.8 and 6.4 mPa·s, respectively), closer to the human tear film physiology.

According to the phosphate content and to the classification system described in the material and methods section, the formulation EDF 3 was categorized as “phosphate-free”; EDF 1, EDF 5 and EDF 9 were categorized as “without phosphates”, and the rest of formulations as “with phosphates”.

### Cell toxicity

The effect of the studied formulations on cell viability was evaluated in HConEpiC and HCEpiC cells after 24- and 72-h exposure to ophthalmic formulations using the MTT reduction assay.

**Figure 2:**
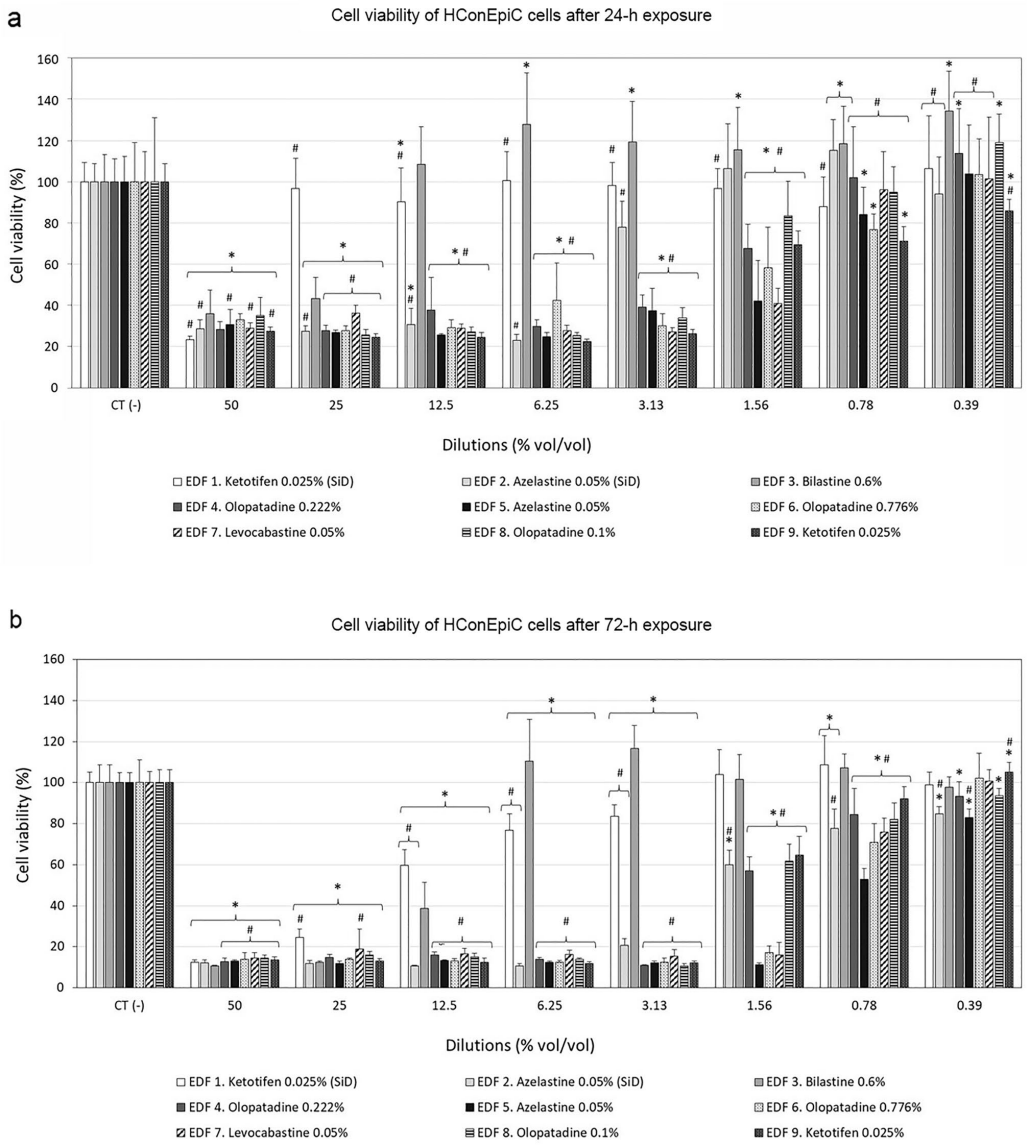
Cell viability of Human Primary Conjunctival Epithelial Cells (HConEpiC) after (**a**) 24-h, and (**b**) 72-h treatment, with serial dilutions of the tested ophthalmic formulations. SiD: single-dose. *p < 0.05 versus control cells; #*p* < 0.05 versus EDF 3 (bilastine 0.6%).

Viability of conjunctival cells (HConEpiC) considerably decreased after 24-h treatment with 50% v/v to 6.25% v/v diluted formulations (Fig. 2a). However, the 12.5% v/v dilution and lower dilutions of the agents EDF 1 and EDF 3 presented the highest survival rates, indicating a low toxicity of these formulations. In contrast, all BAC-containing agents (EDF 4 to EDF 9) showed very high toxicity rates even at 1.56% v/v dilution, and statistically significant differences in relation to EDF 3 (#*p* < 0.05). After 72-h treatment, similar results were obtained (Fig. 2b). Preservative-free bilastine 0.6% eye drops (EDF 3) presented the lowest cell toxicity in comparison to BAC-containing formulations.

**Figure 3:**
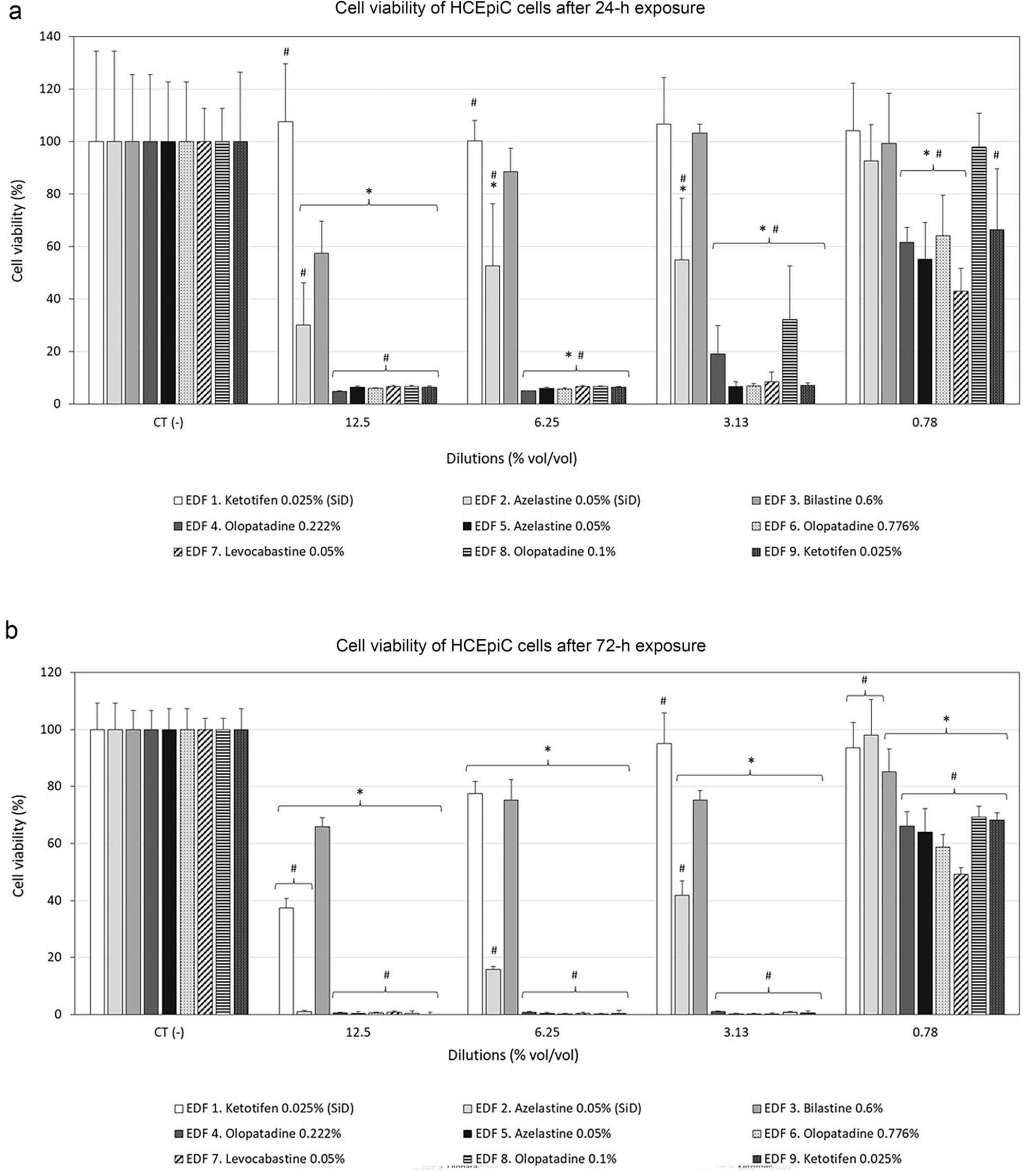
Cell viability of Human Primary Corneal Epithelial cells (HCEpiC) after (**a**) 24-h, and (**b**) 72-h treatment, with serial dilutions of the tested ophthalmic formulations. SiD: single-dose. *p < 0.05 *versus* control cells; #*p* < 0.05 versus EDF 3 (bilastine 0.6%).

The viability of corneal cells (HCEpiC) after 24-h and 72-h exposure to the antiallergic formulations at different dilutions is shown in Fig. 3. In general, EDF 1 and EDF 3 showed the highest cell survival rates, followed by EDF 2. After 24-h treatment (Fig. 3a), EDF 1 did not affect cell viability at any of the tested dilutions, and the survival rates after treatment with EDF 3 were close to 90–100% at 6.25% and lower dilutions. In the case of EDF 2, cell viability increased with decreasing concentrations of the drug, showing survival rates around 50% at both 6.25% and 3.13% dilutions, and above 90% at 0.78% v/v dilution. In contrast, all antiallergic eye drops containing BAC (EDF 4 to EDF 9) significantly reduced cell viability of HCEpiC at all dilutions, with significant differences *versus* EDF 3 (#*p* < 0.05). At 0.78% v/v, cell viability after treatment with EDF 4, EDF 5, EDF6, EDF 7 and EDF 9 was in the range of 40–60%, whereas it was close to 100% for EDF 8. After 72-h exposure (Fig. 3b), cell viability of HCEpiC for all antiallergic eye drops containing BAC (EDF 4 to EDF 9) was drastically reduced at 12.5%, 6.25% y 3.13% v/v (approximately 95% reduction), with significant differences *versus* EDF 3 (#*p* < 0.05). Cell viability for EDF 2 decreased below 5% at 12.5% v/v dilution, whereas for EDF1 and EDF 3 it dropped to values below 40% and 75%, respectively. At 6.25% and 3.13% v/v dilution, EDF 1 and EDF 3 showed survival rates above 75%. After treatment with the lowest dilution of EDF1, EDF 2 and EDF 3, cell viability increased above 80%, whereas it dropped below 70% after treatment with BAC-containing formulations.

### Oxidative stress assessment/ROS production

**Figure 4:**
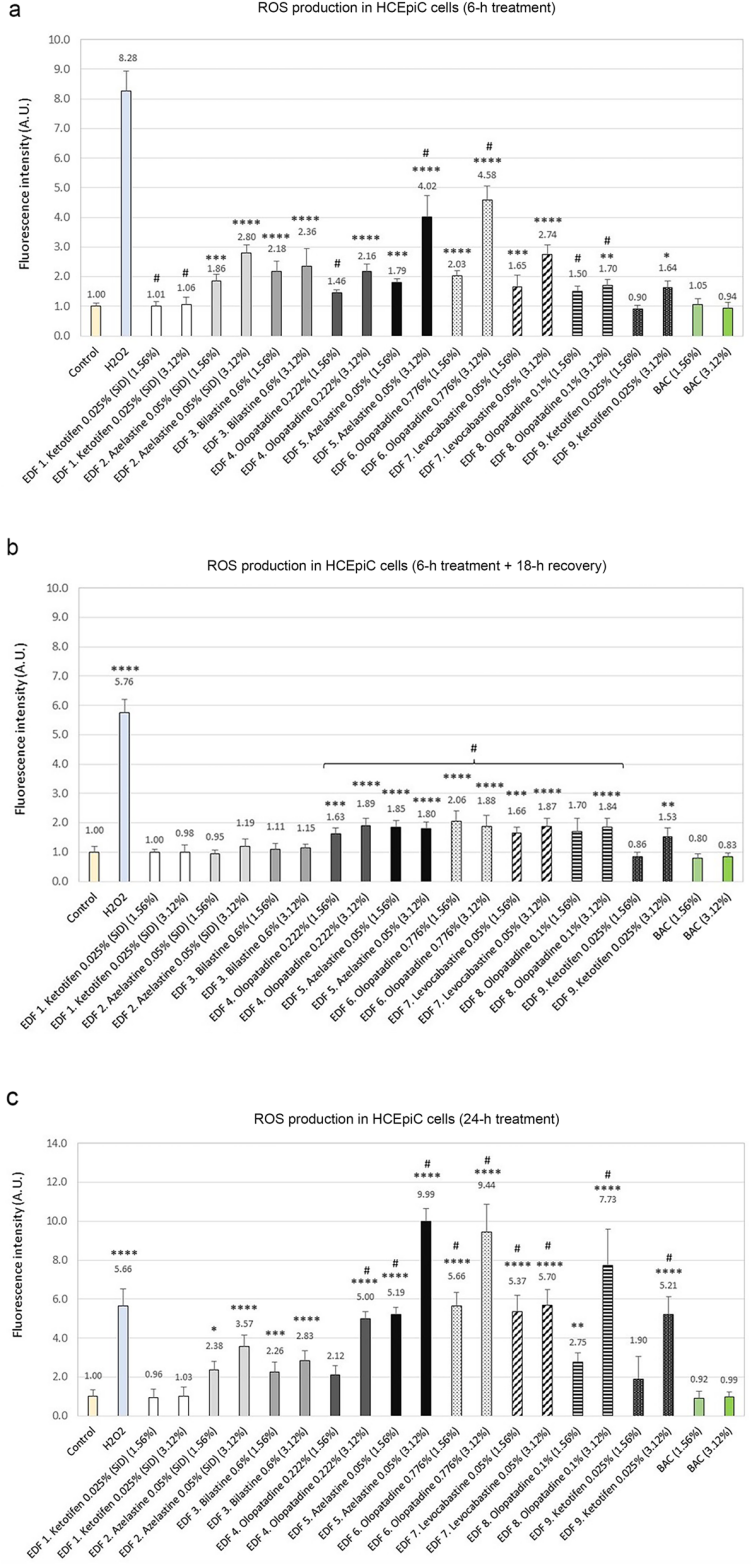
Measurement of reactive oxygen species (ROS) production in Human Primary Corneal Epithelial cells (HCEpiC) in response to treatment with the antiallergic ophthalmic formulations and benzalkonium chloride (BAC). Bars represent CM-H_2_DCFDA fluorescence intensity after (**a**) 6-h treatment, (**b**) 6-h treatment + 18-h recovery, and (**c**) 24-h treatment. Negative control (cells maintained in serum-free medium) and positive control (cells treated with 250 µM H_2_O_2_) were included. The results are shown as normalized fluorescent intensities (mean ± SD). *p < 0.1, **p < 0.01, ***p < 0.001, ****p < 0.0001 *versus* control cells; #*p* < 0.05 versus EDF 3 (bilastine 0.6%).

ROS generation was measured to investigate the ROS-related cytotoxic effect of the ophthalmic preparations (at dilutions 3.12% and 1.56% v/v) in HCEpC cells (Fig. 4), after different exposure times. The experiments showed that all eye drops, except for EDF 1, induced ROS generation in a dose-dependent manner after 6 h of treatment (Fig. 4a). ROS production following treatment with 3.12% dilutions of EDF 5 and EDF 6 showed the highest fluorescence intensity values (4.02 ± 0.70 and 4.58 ± 0.47, respectively). EDF 2, EDF 3, EDF 4 and EDF 7 had a lesser effect, increasing ROS levels 1.5–3 times compared to the control cells. Solutions EDF 8 and EDF 9 slightly increased the production of ROS (less than twofold change), and only at the highest concentration. No effect was observed for BAC formulations at the tested doses (1.56% and 3.12%) under the experimental conditions assayed. After a recovery period of 18 h, a decrease in ROS production levels was observed (Fig. 4b). ROS fell to values close to the control for EDF 2 and EDF 3 at both dilutions, and EDF 9 at 1.56% dilution. ROS levels were still slightly elevated (up to twofold change) for the rest of the products: EDF 4, EDF 5, EDF 6, EDF 7, and EDF 8 at both doses, and EDF 9 at the highest dose (3.12% v/v). There were statistically significant differences between these values and the ROS levels observed for EDF 3 (#*p* < 0.05). After a longer exposure time (24-h treatment) (Fig. 4c), formulations EDF 5, EDF 6, and EDF 8 strongly induced ROS generation at the highest dose, reaching values between almost 8 and 10-times higher than those of the control cells. EDF 5 and EDF 6 at the lower assayed dose, EDF 4 and EDF 9 at the higher assayed dose, and EDF 7 at both doses, produced an increment of ROS around fivefold, comparable to the levels induced by the positive control (cells treated with 250 µM H_2_O_2_). A lower increase of ROS was observed for EDF 2 and EDF 3 at both dilutions. Treatment with EDF 1 at both doses kept ROS production at the same level as the untreated control. No effect of the assayed BAC doses on ROS increase was observed.

### Apoptosis evaluation

**Figure 5:**
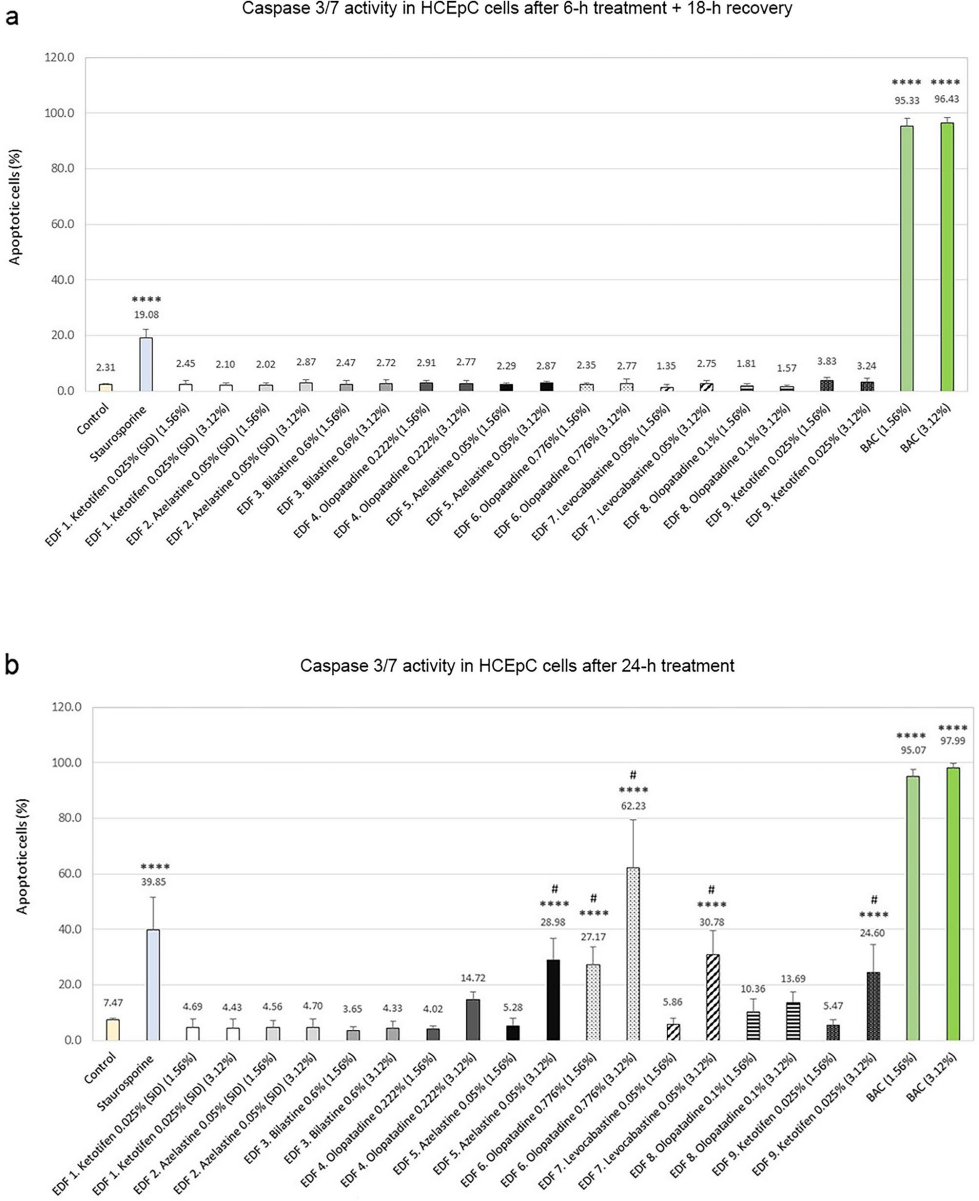
Quantification of caspase 3/7 activity in Human Primary Corneal Epithelial cells (HCEpC) in response to treatment with the antiallergic ophthalmic formulations and benzalkonium chloride (BAC). Bars represent caspase 3/7 fluorescence activity after (**a**) 6-h treatment and 18-h recovery, and (**b**) 24-h treatment. Treatment with 0.5 µM staurosporine was used as internal positive control. *p < 0.1, **p < 0.01, ***p < 0.001, ****p < 0.0001 versus control cells; #*p* < 0.05 versus EDF 3 (bilastine 0.6%).

To explore the apoptotic activity, the caspase 3/7 activity in HCEpC cells in response to 6-h and 24-h treatment with the antiallergic ophthalmic agents was quantified (Fig. 5).

None of the tested products had a substantial effect in caspase 3/7 activation after 6 h of treatment followed by 18 h of recovery (Fig. 5a). Regarding BAC solution, apoptosis was observed in a high percentage of the cells treated with this compound at both dilutions, even above the apoptotic levels induced by the positive control staurosporine. After 24-h treatment, the single-dose formulations (EDF 1 and EDF 2), EDF 3, EDF 4, and EDF 8 did not significantly induce apoptosis. However, the highest dose (3.12% v/v) of EDF 5, EDF 7, EDF 9, and both doses of EDF 6 produced a significant increase in the percentage of apoptotic cells with respect to control (****p < 0.0001) (Fig. 5b). Differences were also significant when compared to EDF 3 (#*p* < 0.05).

**Figure 6:**
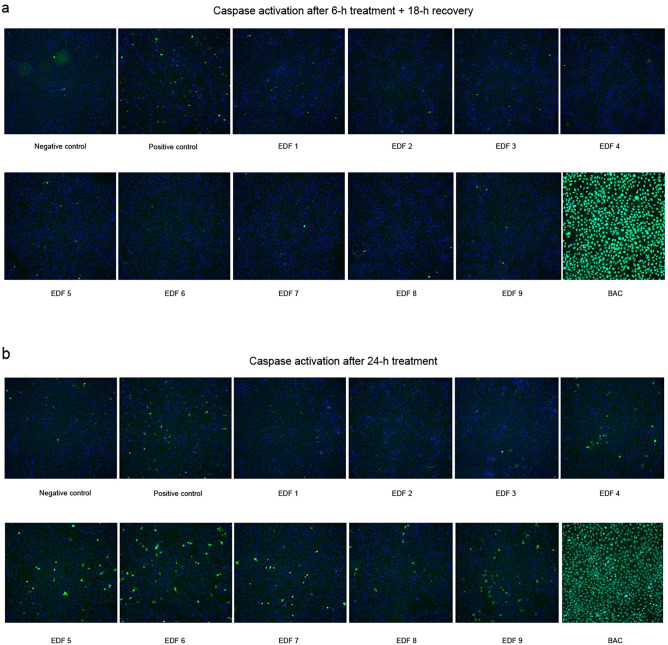
Caspase 3/7 activation and Hoechst nuclear stain after treatment with the antiallergic ophthalmic formulations and benzalkonium chloride (BAC) at the highest dose (3.12% v/v) after (**a**) 6-h treatment + 18-h recovery, and (**b**) Results after 24-h treatment. Images were acquired using CellInsight CX7 High-Content Screening (HCS) Platform (20X objective). Apoptotic cells with activated caspase showed bright green spots, and nuclei were stained in blue. Treatment with 0.5 µM staurosporine was used as positive control of caspase activation.

To further assess the cellular viability, nuclear staining was performed by using the DNA-specific and fluorescent probe Hoechst 33342 dye. Apoptotic cells with activated caspase showed bright green spots, whereas cells without activated caspases showed residual fluorescence signal, and nuclei labeled with Hoechst were stained in blue. Representative pictures of the caspase activation after 6-h and 24-h treatment with the different ophthalmic preparations, and BAC solution at the highest dose (3.12% v/v) are shown in Fig. 6. In the assayed conditions, EDF 1, EDF 2, and EDF 3 did not reduce the number of cells as compared to control cells.

## Discussion

In this work, the physicochemical properties of the new once-daily multidose preservative-free bilastine 0.6% formulation (EDF 3) and the main available commercially preserved and preservative-free eye drops for the treatment of AC were firstly evaluated. The components of topical ophthalmic formulations may alter the composition of the precorneal tear film and induce ocular surface damage when they are used for a long period of time or overdosed^37^. Thus, experimental determinations of pH, osmolarity, viscosity, and phosphate levels of the selected topical ophthalmic formulations were performed to examine whether the physicochemical properties of the formulations analyzed were within the ophthalmic physiological range.

To optimize ocular comfort after instillation, the pH of ophthalmic formulations should match the tear film or at least be in the ocular range of 6.6–7.8, as suggested in several reports^39–41^. In the present study, regardless of the presence of a preservative in their composition, five formulations (EDF 3, EDF 4, EDF 6, EDF 7, and EDF 8) had a pH value within the ocular comfort range, whereas the remaining four (EDF 1, EDF 2, EDF 5, and EDF 9) had acidic pH values (< 6), and would be prone to induce ocular discomfort. On the contrary, it may be assumable that the rest of formulations, which had pH values in the range of 6.94 (for EDF7) to 7.32 (for EDF3), and closer to the physiological tear’s pH (~ 7.4)^33,34^, will not produce initial discomfort. Interestingly, when comparing the mean pH values of preserved (N = 3) (6.59 ± 0.75) versus preservative-free formulations (N = 6) (6.11 ± 1.07), no significant differences were found. These results agree with a previous report, in which 18 artificial tears available in the market were evaluated, and no significant differences in terms of pH were observed between preserved (7.26 ± 0.47) and preservative-free (7.14 ± 0.38) formulations^42^.

The measurement of osmolarity for most ophthalmic formulations provided values between 245 and 331 mOsm/l. These results are in agreement with a previously published work, in which osmolarity of several ophthalmic formulations was measured, and the range of seven antiallergic agents included in the study was 265–341 mOsm/l^33^, similar to the range obtained in this study. As the average normal tear film osmolarity is reported to be around 300 mOsm/l^34–36^, formulations with an osmolarity close to this value could be considered appropriate for the maintenance of ocular homeostasis. In the present study, EDF 3 (294 mOsm/l), EDF 4 (308 mOsm/l), and EDF 8 (295 mOsm/l) showed the closest values to tear film osmolarity. The highest osmolarity was detected in EDF 7 (1,038 mOsm/l), the only ophthalmic drug in the study formulated as suspension, not as solution, showing a mean value far above the physiological tear osmolarity. Topical drops with a hyperosmolar character have been seen to be deleterious to the ocular surface, increasing inflammation and exacerbating tear film instability^33,43,44^.

Viscosity of ocular formulations also plays an important role on drug concentrations in the tear fluid and ocular drug bioavailability, because the drainage rates and the residence time of eye drops depend on the viscosity of the instilled fluids^37^. A high viscosity enables the formulation to remain in the eye longer and gives more time for the drug to exert its therapeutic activity. However, if viscosity is too high, it may reduce tolerability due to blur, stickiness, and build-up of residue on the lids and lashes^45^. In this study, all formulations had viscosity values < 10 mPa·s, except for EDF 6 (27.7 mPa·s). This is relevant, since the rheological properties of the eye drop formulations affect their ability to hydrate, lubricate, and maintain the desired physical and chemical conditions on the corneal surface. Formulations EDF 1, EDF 2, EDF 4, EDF 5, EDF 8, and EDF 9 presented low viscosity values (range of 1.2–3.0 mPa·s), close to the viscosity of water (1 mPa·s)^46^, and at the lower limit of the tear viscosity (~ 1.5 mPa·s)^37^. Therefore, they may be more susceptible to rapid drainage, reducing ocular drug bioavailability. In these cases, multiple instillations would be required to attain longer relief from AC symptoms. On the contrary, formulations with viscosity values above the normal ocular comfort range, such as EDF 6, may reduce tolerability and consequently impact patient compliance and acceptance^37,47,48^. Formulations EDF 3 and EDF 7, on the other side, showed values within the range of the human tear film viscosity (6.8 mPa·s and 6.4 mPa·s, respectively)^38^. In the case of EDF 3, it could probably be due, at least in part, to the presence of the water-soluble polymer hyaluronic acid (HA), which acts as lubricant in the reduction of surface friction and increases precorneal retention time due to its viscous and mucoadhesive properties^49^. In the case of EDF 7, the formulation contains polyethylene glycol and hypromellose, both of which are common ingredients included in ophthalmic formulations for their lubricant and viscosity promoting properties^50^.

The phosphate levels present in the antiallergic agents were also analyzed, since phosphates, which are widely used as part of the buffering system of eye drops, may cause irreversible corneal calcification and visual loss^51^. Therefore, phosphate-free or formulations with low phosphate concentrations close to the physiological levels (≤ 1.45 mmol/l) are preferable^52,53^. According to our classification system, EDF 7 (9,207 ppm), EDF 8 (3324 ppm), and EDF 4 (3213 ppm), followed by EDF 2 (3.66 ppm), and EDF 6 (1.8 ppm) were within the “with phosphates” category. Usually, eye drops with a high concentration of phosphates have the potential to favor the formation of insoluble crystalline calcium phosphate deposits, and should therefore be avoided^52^. The formulations that were classified as “without phosphates” (< 0.6 ppm) were EDF 1, EDF 5, and EDF 9; whereas EDF 3 was the only formulation considered “phosphate-free” (< 0.3 ppm). These results demonstrate the substantial variability in phosphate concentration among the antiallergic formulations under study, that could not be correlated with the presence or absence of BAC, nor to the delivery system of the formulation (single-dose or multidose), as reported previously^54^.

The present study also investigated the cytotoxic effect on conjunctival and corneal epithelial cells of the antiallergic formulations, determined by the MTT assay. In general, all antiallergic eye drops containing BAC (EDF 4 to EDF 9), drastically reduced cell viability of both cell lines at 24 h and 72 h, even at low concentrations. This effect was more substantial in corneal cells and at longer exposure times. In contrast, preservative-free formulations such as EDF 1 and EDF 3, hardly induced cell toxicity when used at 12.5% v/v or lower concentrations. These results show the basic contribution of BAC in the cytotoxic effects induced by antiallergic eye drops, and are consistent with previous studies where cell viability was more affected in BAC-containing antiallergic drugs than in those without the preservative^55–60^. Similarly, ophthalmic agents containing BAC that are used to treat other chronic ocular pathologies have been reported to be more cytotoxic than preservative-free formulations^61,62^. It must be considered that the present cytotoxic experiments were performed for 24 h and 72 h (not for a short-time period) to mimic the potential cumulative effects associated with long-term exposure to unpreserved and BAC-preserved antiallergic agents, as performed in similar studies previously published^58,63^.

To tentatively extrapolate the significance of the in vitro results of this study to those effects expected after in vivo administration, it must be considered that about 5% of the total drug dose administered in eye drops is retained at the ocular surface after instillation, while the excess volume is rapidly washed out via the nasolacrimal drainage system^64,65^. Taking this into account, and considering an average eye drop volume of about 30 µl^66^, the in vitro dilution equivalent to the 5% retained at the ocular surface is 0.78% v/v for the once-daily antiallergic eye drops tested in the present study, namely EDF 3 (bilastine 0.6%), EDF 4 (olopatadine hydrochloride 0.222%) and EDF 6 (olopatadine hydrochloride 0.776%). According to the results obtained, after 24-h incubation, EDF 6 at 0.78% v/v dilution reduced cell viability of conjunctival cells (~ 15% reduction), whereas EDF 3 and EDF 4 maintained cell viability values around 100% at the same dilution. Nonetheless, after 72-h incubation, both EDF 4 and EDF 6 significantly reduced cell viability, especially EDF 6. Since EDF 4 and EDF 6 contain the same API, we hypothesize that the higher toxicity of EDF 6 with respect to EDF 4 could be due to the higher concentration of BAC (0.015% *versus* 0.01%) and/or API (0.776% *versus* 0.222%, respectively) in its composition. In the case of corneal epithelial cells, EDF 4 and EDF 6 exhibited considerable and significant cell toxicity at both time points (~ 35–40% reduction), whereas EDF 3 did not induce or induced a less substantial cytotoxic effect after 24-h and 72-h exposure (0 and ~ 15% reduction, respectively). The recommended dosage regimen for the rest of the studied antiallergic eye drops is twice-a-day (EDF 1 and EDF 9) and four-times-a-day (EDF 2, EDF 5, EDF 7, and EDF 8). Therefore, the in vitro results cannot be directly extrapolated since a repeated dose toxicity study should also be considered.

The present investigation was additionally focused on the effect of the antiallergic eye drops on ROS production and apoptosis in corneal epithelial cells. Oxidative stress plays an important role in the development of different pathologies, including several ocular diseases^67,68^. During environmental stress (such as exposure to chemicals), ROS levels may increase dramatically and cause significant damage to cell structures^69^. In this study, ROS production was investigated in corneal cells after treatment with different concentrations of antiallergic agents. All eye drops, except EDF 1, significantly induced ROS production in a dose-dependent manner. After 6-h treatment, EDF1, EDF 9, EDF 8, EDF 4 and both EDF 2 and EDF 3, in this order, induced low to mild increase in ROS, whereas the highest increments were observed for EDF 5 and EDF 6, which contain the two highest BAC concentrations (0.0125% and 0.015%, respectively). After the 18-h recovery period, ROS levels markedly decreased for almost all formulations. In the case of EDF 2 and EDF 3, ROS fell to values close to the control. After 24-h treatment, ROS production dramatically increased for BAC-containing formulations, specially at higher concentrations. In contrast, EDF 1 did not induce ROS production, and EDF 2 and EDF 3 only induced a mild increase, similar to the values observed after 6-h treatment. All these findings lead to several conclusions. First, treatment with formulations led to an increase in ROS levels in a dose-dependent manner, since 3.12% v/v concentration caused an increase in ROS production as compared to the lower concentration (1.56% v/v), after 6-h and 24-h treatment. Second, after long-term treatment (24 h), the integrity of the ocular surface is less likely to be compromised with the preservative-free formulations EDF 1, EDF 2 and EDF3 than with the BAC-containing agents. This conclusion is also being supported by the fact that EDF 1 (ketotifen 0.025%, single-dose) did not induce ROS production, whereas EDF 9 with the same active ingredient (ketotifen 0.025%, multidose, BAC-containing formulation), especially after 24 h of treatment, did substantially increase ROS production A similar behavior was observed in the case of EDF 2 (azelastine hydrochloride 0.05%, single-dose) and EDF 5 (azelastine hydrochloride 0.05%, multidose). Third, the results also suggest that the oxidative stress in corneal epithelial cells may be caused not only by the preservative but also by the API and/or other excipients in the formulations. In this sense, EDF 1 contains the same excipients as EDF 3 (glycerol, sodium hydroxide and water for injections), except for hydroxypropyl β-cyclodextrin, methyl cellulose, and sodium hyaluronate, only present in EDF 3. Therefore, since both agents are preservative-free formulations, differences between them might be due either to the active ingredient or any of the different excipients present in EDF 3.

The cytotoxic effects of BAC on ocular tissue cells have been extensively documented, and BAC is known to trigger mitochondrial oxidative stress^70,71^. When used in topical ophthalmic formulations, BAC is usually associated with multiple adverse effects on ocular health that are aggravated with chronic exposure^17^. Interestingly, no effect on ROS production was observed for BAC dilutions at the tested doses (1.56% and 3.12% v/v) at any time point, although the in vitro studies carried out so far have shown that BAC increases the formation of ROS in both corneal and conjunctival epithelial cells^72–75^. In one of these studies, a dose-related increase in ROS levels was observed in corneal epithelial cells exposed for 30 min to various concentrations of BAC (0.00005%, 0.0001%, 0.0005% and 0.001%)^72^. Although BAC concentrations tested in the present work are within the same range as the ones used in the mentioned study (3.12% v/v and 1.56% v/v dilutions correspond to 0.0005% w/v and 0.0002% w/v, respectively), ROS production was not detected after 6-h treatment. We hypothesize that, after 6 h ROS production has already occurred, leading to subsequent physiological processes related to, or triggered by ROS activation, ultimately causing cell death^73^.

Despite that reports and experimental studies have repeatedly and consistently focused on the large part played by BAC in the induction of inflammatory ocular surface changes and progressive ocular discomfort upon instillation of eye drops, detrimental effects may also be caused by the API and/or vehicle in the topical ophthalmic formulation^27^. Indeed, it has been described that the decrease of cell viability may also be affected by the different types of antiallergic drugs at short-term exposures and when low concentrations of BAC are present in the formulations^57,60,76^.

The effects of the ophthalmic preparations analyzed on caspase 3/7 activation and apoptosis were minimal when cells were incubated for a short period of time. EDF 3 and the rest of preservative-free formulations did not induce apoptotic cell death at any of the assayed incubation periods. However, apoptosis increased after 24-h treatment for all BAC-containing formulations, being significant for EDF 5, EDF 6, EDF 7, and EDF 9. We cannot rule out a possible protective effect of the API and/or some excipient ingredients of the BAC-containing formulations against BAC, thus retarding activation of apoptosis beyond the 6-h incubation period. In this sense, mannitol, which is present in EDF 6, has been described to exert a protective effect on corneal damage caused by BAC in commercially available timolol maleate eye drops^77^.

Although ROS could not be detected after incubation with BAC dilutions under the experimental conditions assayed, they produced a huge activation of caspases with a concomitant cell death at 6-h and 24-h incubation times. These results are in accordance with previous animal and in vitro studies demonstrating that BAC causes harmful effects to several eye structures, including the tear film, cornea, and conjunctiva, and that the preservative alone is even more harmful than the BAC-containing medication^78^.

In summary, the results of this in vitro study evidenced significant differences in the physicochemical properties of several antiallergic formulations. The findings demonstrate that, among all tested products, the new once-daily multidose preservative-free bilastine 0.6% is the most similar to the human tear film physiology in terms of pH, viscosity, osmolarity and phosphate levels, and support the idea that bilastine 0.6% could be used without generating adverse effects on the ocular surface. This formulation also showed higher survival rates as compared to the other antiallergic multidose eye drops, that could be attributed to the absence of BAC in its formulation. In addition, bilastine 0.6% did not induce caspase-3/7-mediated apoptosis, and only induced a transient oxidative stress in corneal epithelial cells. Considering these experimental data, it is evident that the presence of the preservative BAC in the available commercially multidose eye drops examined is, at least in part, one of the main sources of cytotoxicity.

Overall, these outcomes strongly support the use of the new multidose preservative-free, phosphates free bilastine 0.6% ophthalmic formulation in the treatment of patients suffering from AC contributing to preserve and maintain the integrity of the ocular surface.

## Data Availability

All data generated or analyzed during this study are included in this published article.
